# Comparing Latissimus Dorsi Flap to Implant in Breast Reconstruction Following Mastectomy in Breast Cancer Patients: A Systematic Review

**DOI:** 10.1007/s00266-025-05335-4

**Published:** 2025-10-29

**Authors:** Emma N. Dang, Mehraeel E. Saleh, Ahmed Adham R. Elsayed, Marc D. Basson

**Affiliations:** 1 Present Address: College of Medicine, Northeast Ohio Medical University, Rootstown, Ohio 44272 USA; 2 Department of Surgery, Northeast Ohio Medical University, Rootstown, Ohio 44272 USA; 3 Department of Biomedical Sciences, Northeast Ohio Medical University, Rootstown, OH 44272 USA

**Keywords:** Post-mastectomy, Breast reconstruction, Latissimus dorsi flap, Implant, Breast cancer

## Abstract

**Background:**

Breast cancer is the most common cancer in women worldwide and often necessitates a mastectomy. Many patients choose breast reconstruction (BR) to restore appearance and self-image. Common techniques include implant-based and autologous reconstruction, such as with the latissimus dorsi flap (LDF). This systematic review compares LDF and implant-based reconstructions to guide clinical decision-making and improve patient care.

**Methods:**

This systematic review was conducted in accordance with PRISMA guidelines using PubMed, Cochrane, Web of Science, and VHL databases. Studies were included if they involved breast cancer patients who underwent mastectomy followed by either LDF or implant-based reconstruction. Study quality was assessed using STROBE guidelines.

**Results:**

Out of 785 articles initially identified, 19 articles met the inclusion criteria. LDF and implant-based reconstructions had distinct indications, complication profiles, and long-term outcomes, with LDF reconstruction linked to lower revision rates, more natural aesthetic outcomes, and higher patient satisfaction despite donor site morbidity and more postoperative complications. Implant-based reconstruction had higher rates of revision and lower patient satisfaction, but is less invasive and associated with fewer postoperative complications.

**Conclusion:**

LDF reconstruction is associated with lower revision rates, more natural aesthetic outcomes, and greater patient satisfaction despite higher donor site morbidity and increased postoperative complications. In contrast, implant-based reconstruction offers a less invasive option with fewer complications but is linked to higher revision rates and lower satisfaction. Optimal reconstruction outcomes require an individualized approach that carefully considers patient preferences, oncological factors, and procedural risks to support informed decision-making and enhance quality of life.

- This paper addresses a common clinical decision point in breast cancer care by directly comparing two widely used breast reconstruction techniques: LDF and implant-based reconstruction.
- Evidence-based insight into long-term outcomes, revision rates, and patient satisfaction is provided, which can help clinicians better counsel patients on their reconstruction options.
- This systematic review fills a gap in the literature by synthesizing data across multiple studies to guide future research and improve individualized breast reconstruction planning.

**Level of Evidence III:**

This journal requires that authors assign a level of evidence to each article. For a full description of these Evidence-Based Medicine ratings, please refer to the Table of Contents or the online Instructions to Authors  www.springer.com/00266.

**Graphical Abstract:**

**Supplementary Information:**

The online version contains supplementary material available at 10.1007/s00266-025-05335-4.

**Figure:**
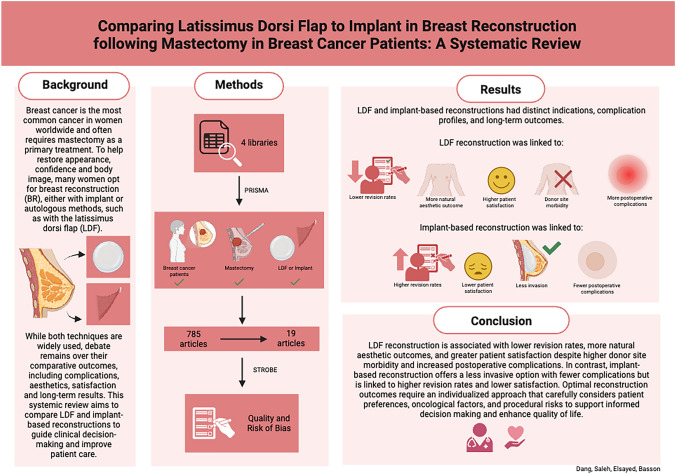
Created in BioRender. Basson, M. (2025) https://BioRender.com/y34x74k

## Introduction

Breast cancer is the most common cancer among women globally and the second leading cause of cancer-related deaths among women worldwide [1]. Globally, in 2022, around 2.3 million women were diagnosed with breast cancer, and about 670,000 died from it [2]. Breast cancer can develop in women of any age after puberty, no matter where they live, but it becomes more common with age [2]. The primary treatment of breast cancer is surgery [2]. In addition to surgery, other treatments like CT (chemotherapy), hormonal therapy, and RT (radiotherapy) can also be used [2]. For patients undergoing mastectomy, the prospect of a potentially disfiguring surgical outcome following a cancer diagnosis can profoundly influence treatment choices and pose significant challenges to psychosocial recovery [3]. Breast reconstruction helps restore body image, leading to improved self-esteem and reduced feelings of depression and anxiety. Women who opt for breast reconstruction report higher satisfaction with their bodies and better overall quality of life, regardless of the timing of the reconstruction [4, 5]. The two most commonly used methods for breast reconstruction are implant-based reconstruction and autologous breast reconstruction. There are various techniques, such as the latissimus dorsi flap (LDF), deep inferior epigastric perforator (DIEP), and transverse rectus abdominis musculocutaneous (TRAM) flaps. This systematic review aims to compare the latissimus dorsi flap (LDF) to implant-based reconstruction following mastectomy.

An implant is a prosthesis that can be positioned either in front of the chest muscle (prepectoral reconstruction), provided the mastectomy skin flap is sufficiently thick, or behind the muscle (submuscular reconstruction) [6]. Multiple implant types are available for breast reconstruction, including cohesive silicone gel, highly cohesive gel (“gummy bear”), saline, and structured saline implants, each offered in a range of surface textures and anatomical shapes to accommodate patient-specific needs [7]. The LDF is a muscle that stretches from the mid to the lower spine, with its pedicle base in the axilla, remaining attached to its original blood vessels, and is moved to the chest to help rebuild the breast shape after a mastectomy [7, 8]. The LDF can be used as a standalone flap or alongside a breast implant to achieve the desired breast size and shape [9].

Despite extensive research, the comparative outcomes of latissimus dorsi flap (LDF) and implant-based breast reconstruction remain unclear, particularly in terms of surgical complications, quality of life, aesthetic results, long-term functionality, patient satisfaction, and durability. It should be noted that while both breast reconstruction types may have differing indications, some patients may be suitable for both types. This difference in indication may partly account for the variation in outcomes reported. This systematic review aims to comprehensively compare these outcomes by synthesizing existing literature to provide evidence-based insights that can guide clinical decision-making, enhance patient care, and serve as a foundation for future research.

## Methods

### Search Strategy

This systematic review was conducted in compliance with PRISMA guidelines. The data were obtained using four databases: PubMed, Cochrane, Web of Science, and VHL. The main keywords used were latissimus dorsi flap, implant, mastectomy, breast cancer, and their relevant synonyms or MeSH terms if available (Supplementary Table 1). Two independent reviewers screened the articles for relevance using title and abstract, followed by a full-text review based on the research question, which compares the latissimus dorsi flap (LDF) and implant in breast cancer patients undergoing breast reconstruction post-mastectomy, and the eligibility criteria. Any disputes were resolved by consensus, and if unresolved, the senior author was consulted.

### Eligibility Criteria

The inclusion criteria were studies involving female patients diagnosed with breast cancer who had undergone mastectomy, and studies in which patients had received breast reconstruction using either LDF or breast implants. Both techniques must have been investigated in the same article to be considered for inclusion.

Our exclusion criteria included articles that did not investigate both techniques but reported either LDF or implant-based reconstruction alone. Furthermore, studies that evaluated LDF without implants and implant-based reconstruction alone were included, while studies that combined LDF with implants were excluded. In addition, articles involving breast reconstruction for non-cancer-related indications were excluded. Non-human studies, such as animal studies and cadaveric studies, were not included. In addition, non-original research, such as reviews, editorials, and commentaries, was excluded. Any articles compiling the total number of populations without indicating the exact number of patients for each technique were also excluded.

### Data Extraction

After completion of the search and filtration process, relevant articles were exported into Zotero, in which the relevant demographic and characteristic data were extracted. This included study design, total population, relevant population, which included patients within the study that are relevant to the research question, age of participants, population characteristics, staging of breast cancer prior to mastectomy, and study relevance. The population characteristics which was the inclusion criteria for the patients to be included in this study. Outcome data were also extracted, including indication, contraindication, surgical technique, postoperative complications, risk factors, aesthetic outcomes, long-term functional outcomes, durability, psychological impact, revisions, and mortality

### Aggregated Calculated Incident

The Aggregated Calculated Incidence (ACI) of postoperative complications, long-term functionality, durability, and revision procedures was reported as percentages. For each incidence, events were categorized based on whether they occurred in the LDF or implant-based reconstruction group. The total number of events within each group was summed across all studies reporting that outcome, then divided by the total number of patients in that same group across all studies that reported such events. This method ensured that incidence rates were accurately calculated based on the number of patients at risk within each reconstruction group.

### Statistical Analysis

Postoperative complications, failures, or losses, and revision rates were compared between the LDF and implant-based reconstruction groups using a two-proportion z-test. For each group, the total number of complications and the total number of patients within each group were summed across all relevant studies. The pooled proportion was calculated as the total number of post-operative complications in both groups divided by the combined sample size. The standard error (SE) of the difference in proportions was then computed using the pooled proportion. The z-score was calculated as the difference in group complication rates divided by the SE. A two-tailed p-value was derived from the z-score using the standard normal distribution. A p-value of less than 0.05 was considered statistically significant. All calculations were performed using Microsoft Excel.

### Reporting Quality Assessment

The authors evaluated the quality and risk of bias for all 19 studies based on their design, following the Enhancing Quality and Transparency of Health Research (EQUATOR) guideline [10–28]. For observational studies, the Strengthening the Reporting of Observational Studies in Epidemiology (STROBE) guidelines were used to assess items such as the title, abstract, introduction, methods, results, discussion, and other information. A color-coding system was used for clarity: green indicated that an item was fully addressed, red signified that it was not clearly addressed, and yellow was used when it was unclear whether an item was addressed or not.

## Results

### Search Strategy

**Fig. 1:**
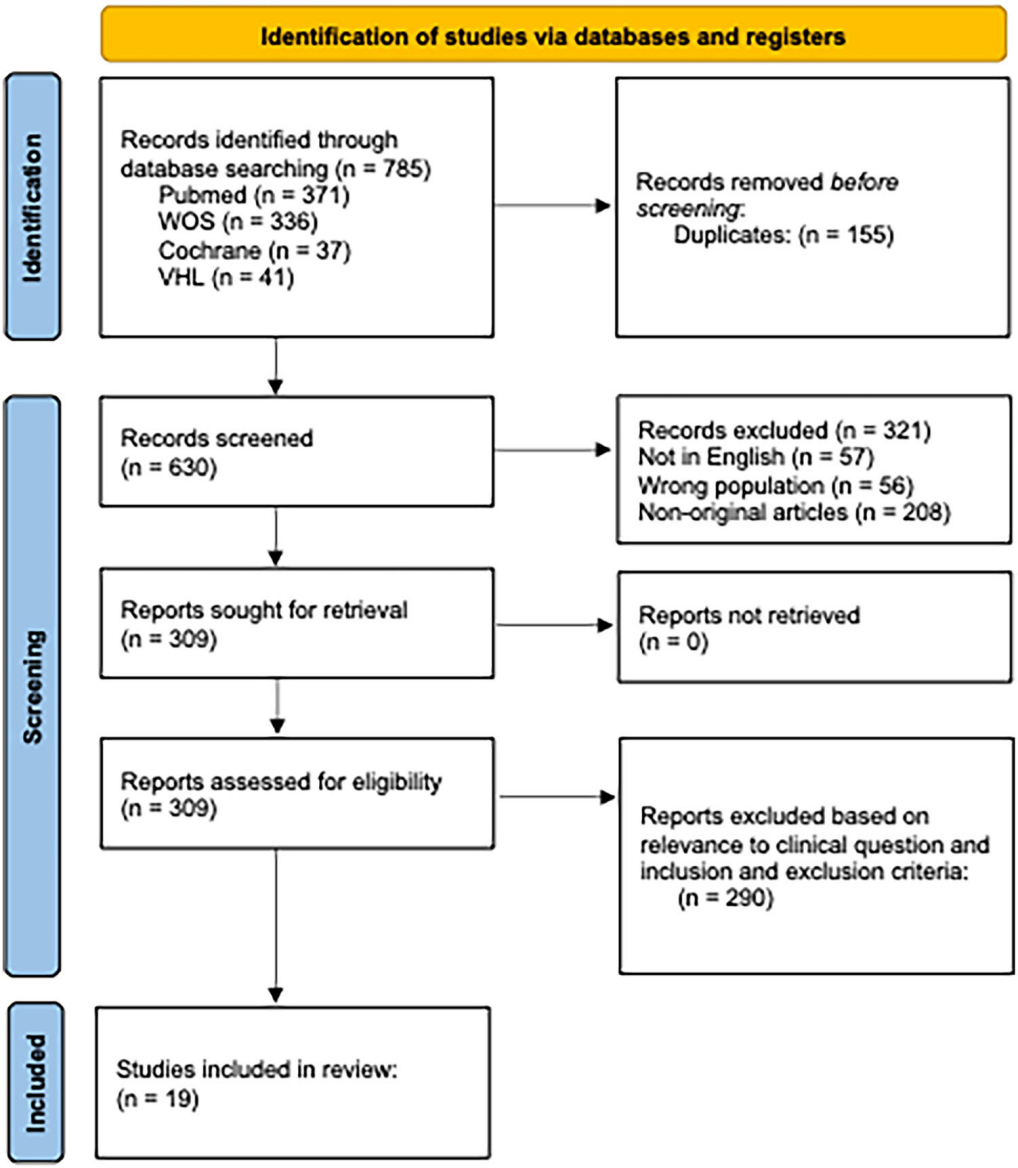
PRISMA flow diagram for new systematic reviews

The search strategy yielded 785 potential articles. After the removal of 155 duplicates, the remaining number was 630. After abstract and title filtration, 321 articles were removed, resulting in 309 articles. After filtration based on relevance to the research question and the exclusion/inclusion criteria, 290 articles were removed. The final number was 19 relevant articles that were included in this systematic review [10–28] (Fig. 1).

### Demographic and Characteristics

Out of the 19 studies, the earliest article by year was published in 1996 [28], and the most recent article was published in 2024 [11]. All 19 articles were observational studies with 11 retrospective cohort studies [10, 11, 14–18, 20, 22, 26, 27], 4 retrospective case series [13, 19, 21, 28], 2 retrospective cross-sectional studies [24, 25], and 2 prospective cohort studies [12, 23]. The largest total population was 16,897 patients [16], and the smallest total population was 32 patients [11]. The largest relevant total population was 7,566 patients [16], and the smallest relevant total population was 17 patients [28]. Of the given relevant population undergoing latissimus dorsi flap (LDF) reconstruction, the largest population was 2,373 patients [16], and the smallest population was 1 patient [13, 23]. Of the given relevant population undergoing implant-based reconstruction, the largest population was 5,193 patients [16], and the smallest population was 10 patients [19]. The highest reported average age of total patients was 70 years old [13], and the lowest reported age was 37 years old [28]. Population characteristics for all the studies varied widely. Staging of breast cancer prior to mastectomy also varied, with some articles providing overall breast cancer stage, tumor grade, and/or nodal involvement (Table 1).

**Table 1:** Demographics and characteristics

Author, year of publication	Study design	Total population	Relevant population	Age of participants	Population characteristics	Staging of breast cancer prior to mastectomy
Allweis et al., 2002 [10]	Retrospective cohort study	357	Total: 21 LDF: 3 implant: 18	Total: 45.8	Post-mastectomy patients undergoing immediate breast reconstruction followed by CT, and post-mastectomy patients without breast reconstruction followed by adjuvant CT.	Stages I-IIIA
Asal et al., 2024 [11]	Retrospective cohort study	32	Total: 32 LDF: 18 implant: 14	LDF: 52.5 ± 8.95 implant: 49.79 ± 3.83	Post-mastectomy female patients undergoing immediate breast reconstruction with an LDF or immediate implant exchange after implant complications.	–
Bennett et al., 2018 [12]	Prospective cohort study	2343	Total: 1596 LDF: 71 implant: 1525	Total: 49.5 ± 10.1 LDF: 53.5 ± 9.7 implant: 48.4 ± 10.3	Post-mastectomy female patients, 18 years and older, presenting for first-time breast reconstruction.	–
De Lorenzi et al., 2010 [13]	Retrospective case series	63	Total: 50 LDF: 1 implant: 49	Total: 70	Post-breast cancer treatment patients, 65 years or older, undergoing immediate breast reconstruction.	Stages T1-T3 and N0-N3
Ditsch et al., 2013 [14]	Retrospective cohort study	139	Total: 35 LDF: 11 implant: 24	Total: 48.7	Post-mastectomy patients treated with breast reconstruction surgery.	Stages T1-T4
Gao et al., 2022 [15]	Retrospective cohort study	135	Total: 135 LDF: 56 implant: 79	LDF: 40.34 ± 9 MLDF group: 38.63 ± 7.75	Post-mastectomy Chinese patients, 18 years and older, presenting for first-time breast reconstruction.	–
Johnson et al., 2023 [16]	Retrospective cohort study	16,897	Total: 7,566LDF: 2,373 implant: 5,193	LDF: 53.3 ± 9.9 implant: 52.9 ± 10.6	Post-mastectomy female patients, 16 years and older, undergoing immediate breast reconstruction.	–
Lei et al., 2020 [17]	Retrospective cohort study	309	Total: 272LDF: 46 implant: 226	–	Post-mastectomy patients with breast cancer undergoing one-stage immediate breast reconstruction.	Stages 0-II
Lipa et al., 2003 [18]	Retrospective cohort study	81	Total: 50 LDF: 24 implant: 26	Total: 69.2 ± 3.3	Post-mastectomy patients, 65 years or older, undergoing breast reconstruction of post-mastectomy defects.	Stages 0-III
Liu et al., 2020 [19]	Retrospective case series	155	Total: 40 LDF: 30 implant: 10	Total: 46.5	Post-mastectomy female patients undergoing breast reconstruction.	–
Mazard et al., 2024 [20]	Retrospective cohort study	155	Total: 155 LDF: 92 implant: 63	Total: 50 ± 10.12 LDF: 50 ± 9.93 implant: 49.6 ± 10.45	Post-mastectomy patients, 18 years or older, undergoing breast reconstruction.	Stages T1-T4
Missana et al., 2007 [21]	Retrospective case series	69	Total: 30 LDF: 5 implant: 25	Total: 51	Patients undergoing autologous fat injections to improve cosmetic breast reconstruction surgery.	–
Quilichini et al., 2020 [22]	Retrospective cohort study	748	Total: 699 LDF: 148 implant: 551	Total: 49	Post-mastectomy patients undergoing immediate breast reconstruction.	–
Reefy et al., 2010 [23]	Prospective cohort study	127	Total: 52 LDF: 1 implant: 51	Total: 47	Post-mastectomy female patients with early-stage breast cancer undergoing immediate breast reconstruction.	Stages T1-T3 and N0-N1
Sanguinetti et al., 2016 [24]	Retrospective cross-sectional study	40	Total: 33 LDF: 12 implant: 21	Total: 46.58 ± 10.59 LDF: 48 ± 12.37 implant: 45.81 ± 11.04	Post-mastectomy female patients with breast cancer undergoing breast reconstruction.	–
Tomita et al., 2023 [25]	Retrospective cross-sectional study	1001	Total: 721 LDF: 117 implant: 604	Total: 54.9 ± 9.1 LDF: 51.4 ± 8.4 implant: 55.3 ± 9.5	Post-mastectomy female patients, 20 years or older, undergoing breast reconstruction.	–
Woo et al., 2018 [26]	Retrospective cohort study	430	Total: 267 LDF: 44 implant: 223	Total: 43 ± 7.4 LDF: 41.6 ± 6.6 implant: 42 ± 7.8	Post-mastectomy patients undergoing immediate breast reconstruction.	Stages T0-T3
Xia et al., 2023 [27]	Retrospective cohort study	91	Total: 91 LDF: 52 implant: 39	–	Post-mastectomy female patients undergoing immediate breast reconstruction.	Stages 0-III
Zekri et al., 1996 [28]	Retrospective case series	52	Total: 17 LDF: 3 implant: 14	Total: 37	Chinese breast cancer patients undergoing immediate breast reconstruction.	-

*CT* chemotherapy, *LDF* latissimus dorsi flap

### Summary of Outcomes

Indications for LDF reconstruction varied widely, including large tumors, higher BMI, failed implants, neoadjuvant radiotherapy (RT), local recurrence, and foreign body concerns. In contrast, indications for implant-based reconstruction included lower body mass index (BMI), bilateral breast reconstruction, and T1-T2 tumors. Contraindications for implant-based reconstruction were diverse as well, including preoperative RT, adjuvant RT, and sentinel lymph node metastasis [13, 19, 22, 24, 27]. Immediate breast reconstruction is almost never recommended in cases when a significant amount of native breast tissue is excised during mastectomy, limiting the viability of a single-stage procedure and increasing potential risks [19]. Techniques used across the included studies differed greatly.

Post-operative complications of both LDF and implant-based reconstruction varied substantially. Shared complications included seromas, wound dehiscence, nipple-areolar complex (NACx) necrosis, flap necrosis, infections, hematomas, delayed scarring, neurological issues, and pneumothorax. Complications unique to implant-based reconstruction included capsular contractures, implant malpositioning, cystosteatonecrosis, and implant leakage. Risk factors for each reconstruction type were also highly variable. Many risk factors overlapped between both LDF and implant-based reconstruction, including smoking, older age, NACx conservation, intrinsic difficulty of the procedure, late rehabilitation, axillary lymph node dissection (ALND), and intraoperative bleeding volume.

LDF reconstruction generally yielded more natural and durable aesthetic outcomes compared to implants, contributing to higher long-term patient satisfaction. Implant-based approaches are more prone to long-term issues such as visible folds, volume concerns, and asymmetry.

Regarding psychological impact, LDF reconstruction was associated with an overall higher satisfaction rate than implant-based reconstruction. Implant-based reconstruction can lead to higher stress and worse psychological adjustment for patients, depending on complications that may occur. However, implants may also be less burdensome and painful. Despite these differences, both breast reconstruction types may provide patients with satisfaction post-mastectomy as their purposes are to restore the appearance of the breast. LDF reconstruction was also less likely to require revision surgery than implant-based reconstruction. Of the reported mortalities, there were only 2 deaths overall as a result of metastatic breast cancer and lung cancer. Neither appeared related to the reconstruction. Cancer recurrence was not influenced by breast reconstruction type (Supplementary Table 2).

### Reporting Quality Assessment

All 19 observational studies were assessed using STROBE (Table 2). None of the included studies reported on the methodology participants section (6b). The majority of the studies adequately reported the following key elements: title and abstract (1b), background/rationale (2), objectives (3), study design (4), setting (5), participants (6a), variables (7), data sources/measurements (8), quantitative variables (11), statistical methods (12a, 12b), participants (13a), descriptive data (14a), outcome data (15), key results (18), limitations (19), interpretation (20), and generalizability (21). However, elements such as title and abstract (1a), bias (9), study size (10), statistical methods (12c, 12 d, 12e), participants (13b, 13c), descriptive data (14b), and main results (16b, 16c) were frequently unclear or unaddressed. Overall, most of the articles adhered to the STROBE guidelines, but there was some lack of clarity in certain methodological items, making the overall reporting quality suboptimal.

**Table 2:**
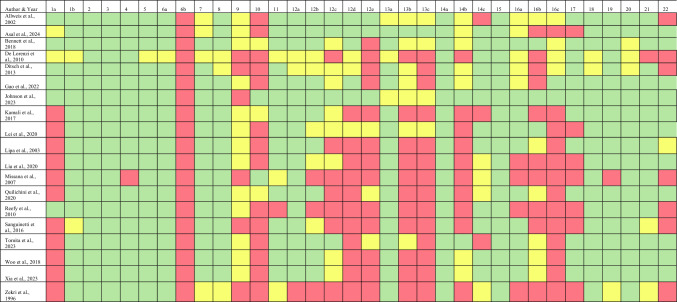
Quality and assessment using STROBE guidelines

Green = item on checklist clearly addressed, Yellow = item on checklist not clearly addressed, Red = item on checklist clearly not addressed

1a = study design in title or abstract; 1b = summary in abstract; 2 = background; 3 = objectives and hypotheses; 4 = study design; 5 = setting; 6 = participants; 7 = variables; 8 = data sources/measurement; 9 = bias; 10 = study size; 11 = quantitative variables; 12a = statistical methods; 12b = methods used for subgroups; 12c = address missing data; 12 d = address loss of follow up in cohort studies, matching cases and controls in case-control studies, and analytical methods in cross-sectional studies; 12e = sensitivity analyses; 13a = number of individuals at each stage of study; 13b = non-participation; 13c = flow diagram; 14a = characteristics of study participants; 14b = number of participants with missing data; 14c = follow-up time in cohort studies; 15 = number of outcome events in cohort and cross-sectional studies, and numbers in exposure category in case-control studies; 16a = unadjusted estimates; 16b = category boundaries; 16c = estimates of relative risk into absolute risk; 17 = other analyses; 18 = key results; 19 = limitations; 20 = interpretation; 21 = generalizability; 22 = funding

## Discussion

### Indications

Large tumors or cancer diagnoses requiring mastectomy are an indication for latissimus dorsi flap (LDF) reconstruction when there is a strong desire to conserve the breast, as the goal of this breast reconstruction type is to reduce the magnitude of defects and complications while maximizing soft tissue coverage provided by the LDF [15, 20]. LDF reconstruction is typically performed following mastectomies for cancer diagnoses to allow for adjuvant treatments, which are better tolerated with autologous tissue due to its ability to protect and nourish skin flaps [20, 22]. Autologous tissue is also more resistant to the damaging effects of radiation compared to implants [15, 18, 22]. Local recurrences requiring further treatment are commonly managed with LDF reconstruction for the same reasons [20]. Additionally, the LDF can fill tumor cavities left by large tumors, providing both structural and aesthetic benefits [15]. Patients with foreign body concerns may prefer LDF reconstruction as it uses the patient’s own tissue, such as the transverse or oblique latissimus dorsi (LD), avoiding the use of synthetic materials [11, 28]. Body mass index (BMI) also significantly influences breast reconstruction choice. Higher BMI is an indication for LDF reconstruction as additional tissue in the LD area provides greater volume for autologous reconstruction, and diabetic patients with higher BMI may benefit from the lower complication rates associated with LDF reconstruction [11, 17, 20, 22]. Breast size correlates with these BMI trends, where larger breasts often accompany higher BMI, favoring LDF reconstruction.

Implant-based reconstruction is often indicated in cases of bilateral breast reconstruction, as implants are frequently used to achieve symmetry by balancing the contralateral breast, making it easier to match size and shape without depending on native tissue volume [18, 23, 29]. T1-T2 tumors in patients with small to medium breasts are also indications for implant-based reconstruction, provided there is adequate skin and an intact pectoralis major (PM) muscle to support implant placement [28]. Early-stage, low-grade tumors may not require adjuvant chemotherapy (CT), making implant-based reconstruction a suitable option [10]. Tissue expanders (TE) may be used to accommodate limited skin in such cases [20]. Implants are often positioned above or below the PM muscle, requiring its integrity for successful outcomes [11, 15, 20, 23, 25, 26]. Lower BMI is also an indication for implant-based reconstruction due to insufficient donor site volume, which limits the feasibility of autologous flaps in leaner patients [15, 17–20, 28]. However, small to medium breasts can be addressed with either method, depending on additional factors [15, 17–20, 22, 28]. Age can also play a complex role, with implant-based reconstruction sometimes preferred in older patients as a less disruptive option despite a higher risk of complications due to the reluctance of both surgeons and patients to undergo the longer operative times associated with autologous procedures [13, 28]. However, implants can also be the first choice in younger patients when appropriate, with the shared goal of restoring the breast’s appearance regardless of age [13].

LDF reconstruction is frequently recommended following failed implant reconstruction, which is a scenario not commonly reversed [18]. This approach offers a reliable option for patients who have experienced complications with implants, allowing for a safer reconstructive solution with reduced risk of additional complications and shorter durations of postoperative care [11].

#### Contraindication

While higher BMI is an indication for LDF reconstruction, patients classified as class III obese may have an increased risk for postoperative complications due to impairment of immune responses and increased susceptibility to delayed wound healing as a result of compromised blood flow [30]. Though conflicting evidence of elevated BMI being a risk in LDF reconstruction exists, the clinical context of these patients undergoing breast reconstruction should be fully considered [30]. Additionally, flap size is an important consideration when evaluating the appropriateness of LDF for breast reconstruction. Depending on the patient, there may be a volume discrepancy, and the donor site may not be sufficient for the size of the breast required for reconstruction [20]. In contrast, use of a large LDF may require wider dissection of the donor site, which creates a larger space at risk of seroma formation or wound complications [29].

**Fig. 2:**
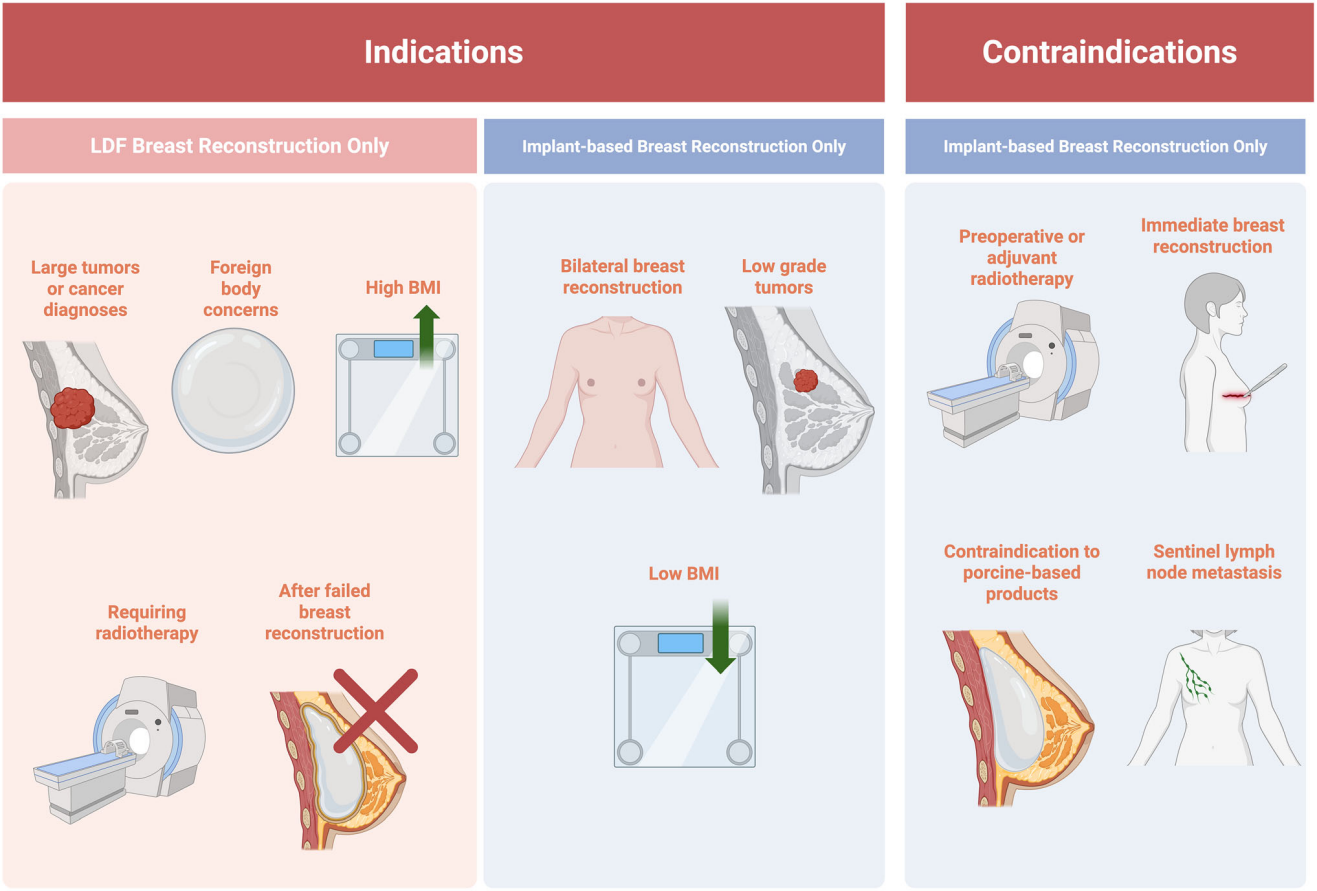
Indications and contraindications for LDF and implant-based breast reconstruction. Created in BioRender. Basson, M. (2025) https://BioRender.com/fgf9ad9

Contraindications for implant-based reconstruction include preoperative or adjuvant RT, as radiation has a detrimental effect on implant outcomes, increasing complication rates and compromising cosmetic results [13, 19, 22, 24]. A potential strategy for implant-based reconstruction after post-mastectomy radiotherapy (PMRT) involves immediate placement of a TE followed by delayed reconstruction with either an implant or LDF, mitigating the adverse effects of RT on cosmetic outcomes [23]. Immediate breast reconstruction with implants is also contraindicated when a significant amount of native breast skin must be excised during mastectomy, limiting the feasibility of single-stage implant placement [19]. Although direct-to-implant (DTI) reconstruction with biological matrices can improve surgery time and resource use, patient-reported outcomes have been found to favor immediate LDF reconstruction in some cases [15, 31]. Additionally, contraindications to porcine-based products excluded patients from procedures involving porcine small intestine submucosa (SIS) matrix-assisted DTI reconstruction, as allergies or objections to porcine materials make this option unsuitable [15]. Sentinel lymph node metastasis is another contraindication for implants, as the need for subsequent therapies increases the risk of implant-related complications [27] (Fig. 2).

### Technique

**Fig. 3:**
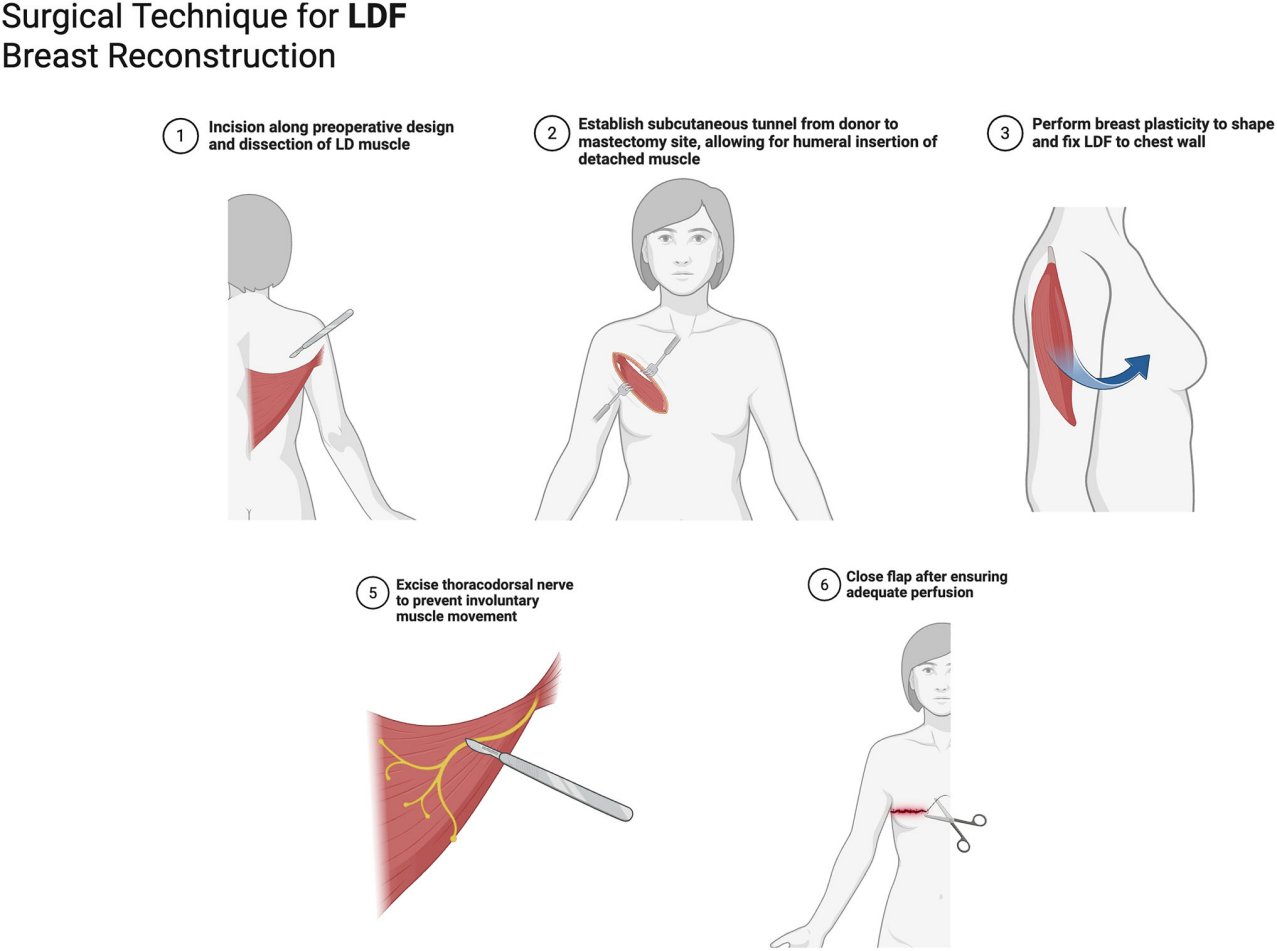
Surgical technique for LDF breast reconstruction. Created in BioRender. Basson, M. (2025) https://BioRender.com/4jhnzyc

The surgical process of LDF reconstruction begins with incisions made along the preoperative design, followed by dissection of the LD muscle and overlying adipose tissue toward the scapula [11]. Flap selection depends on available tissue and desired scar placement [32]. A transverse LDF flap is often chosen to hide the scar along the bra line, while an oblique flap creates a V-shaped open-back appearance [32]. Beyond the traditional myocutaneous flap, options include endoscopically harvested muscle-only flaps, which are used to minimize scarring when a skin paddle is unnecessary, and extended LDFs, which recruit additional tissue to increase flap volume [18, 33, 34]. A subcutaneous tunnel is created to connect the donor site to the mastectomy site, allowing the flap to be transferred and fixed to the chest wall with humeral insertion of the detached muscle [26, 27, 35]. Breast plasticity techniques are then employed to contour the flap for a natural appearance, and the thoracodorsal nerve is excised at a 1 cm width to prevent involuntary muscle contraction [27, 36]. Two suction drains are placed in the mastectomy and donor sites to evacuate fluid and reduce seroma formation [26, 27, 37]. The flap is closed after ensuring adequate perfusion, as ischemia can result in necrosis or flap loss [27, 38] (Fig. 2). Adipose tissue transfers may be incorporated for improved cosmetic outcomes in breast shape and neckline [20]. These are typically performed via atraumatic liposuction or Coleman’s lipoinjection procedure involving harvesting adipocytes, purifying them through centrifugation, and reinjecting them as needed [21]. When LDF reconstruction is performed simultaneously with the mastectomy or subcutaneous gland resection, careful preservation of thin tissue layers around the areola maintains nipple blood supply while maximizing breast skin preservation and achieving tumor-free margins [27, 39]. Additionally, robotic-assisted LDF reconstruction may be employed to improve surgical exposure, minimize incision size, reduce tissue trauma, and enhance flap viability [22] (Fig. 3).

The process of implant-based reconstruction begins with preparing the implant based on patient anatomy and breast size requirements [40]. When implant-based reconstruction is performed alongside mastectomy or subcutaneous gland resection, the process can be very similar to LDF reconstruction as described previously [28]. Additionally, a single-stage DTI procedure or a two-stage approach with initial TE placement followed by implant exchange may be performed [10, 12–14, 16, 18–20, 28, 41]. A TE is often selected when the skin envelope is insufficient, allowing gradual expansion to reduce tissue damage. Immediate implant placement offers fewer surgeries and shorter reconstruction times [20, 42]. Implants may also vary in shape and size, including anatomically shaped implants, which provide enhanced lower pole projection and upper pole fill with improved rupture resistance, and round-shaped implants, which offer firmness and lower malposition risk [13, 40]. The PM fascia is preserved but then partially stripped to remove most of the PM origin, maintaining the natural curvature of the lower and lateral breast poles, and creating an implant pocket [27, 43]. A drainage tube is inserted into the pre-pectoral or sub-pectoral spaces to prevent fluid accumulation [26, 44]. Implant placement can be either prepectoral or subpectoral, with prepectoral reducing chest wall pain, movement restriction, and capsular contracture risk [45]. But it also carries higher chances of implant visibility, rippling, wound dehiscence, and exposure due to limited tissue coverage [45]. Subpectoral placement requires PM dissection, which can lead to pain, restricted movement, and breast deformity from muscle contraction [15, 45]. However, it also provides superior soft tissue coverage with lower risks of infection, flap necrosis, capsular contracture, and implant exposure or displacement [45]. While subpectoral techniques were originally established to account for the downfalls of early prepectoral techniques, advancements have allowed prepectoral techniques to overcome these challenges [45]. Now, many studies have demonstrated that prepectoral implants are a safe modality that has similar outcomes to subpectoral implants with significantly lower rates of capsular contracture [46]. After implant positioning, the PM fascia is sutured and the incision closed [27]. If PM muscle coverage is insufficient, a porcine SIS or acellular dermal matrix can be secured to the chest wall to reinforce the implant pocket and support the lower and lateral breast areas [15, 26]. Adipose tissue transfers, using the same lipoinjection technique described for LDF, may also be performed to enhance cosmetic results [20, 21].

### Post-Operative Complications

**Fig. 4:**
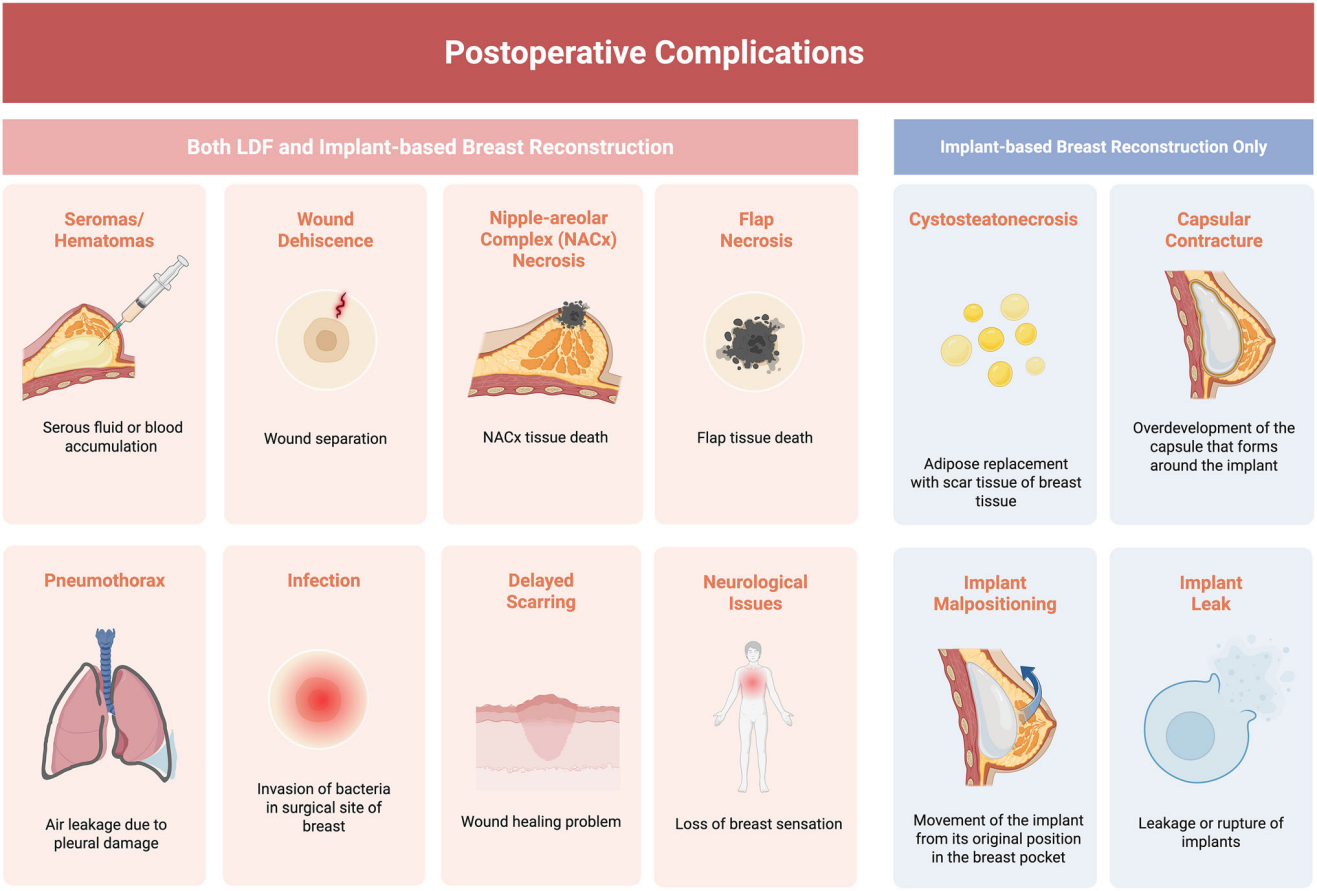
Postoperative complications in LDF and implant-based breast reconstruction. Created in BioRender. Basson, M. (2025) https://BioRender.com/cj5waxq

In the LDF reconstruction group, the aggregated calculated incidence (ACI) of post-operative complications was 26.8% of the cases. In the implant-based reconstruction group, 23% of the cases had post-operative complications [11–13, 15, 18, 20–23, 27]. This complication was not significant (*p* = 0.075). Autologous breast reconstruction is generally considered to have increased morbidity in donor site complications and longer operative times due to the additional donor site [38, 47] (Fig. 4).

#### Seromas

In the LDF reconstruction group, the aggregated calculated incidence (ACI) of seromas was 18.3% of the cases, while 4.2% of the implant-based reconstructions experienced this complication [11, 15, 20–23]. The total number of seromas across all studies was added and divided by the total number of patients within each reconstruction type from the studies in which seromas occurred. The rate of seromas was significantly higher in the LDF reconstruction group compared to the implant group (*p* = 0.000000015). Seromas are a common postoperative issue following breast reconstruction, particularly in LDF procedures [24, 26]. This increased risk in LDF reconstruction arises from the wide dissection required to harvest the LDF, which creates a large dead space at the donor site prone to fluid accumulation [25]. In LDF reconstruction, seromas can develop both at the donor site where the flap is harvested and at the recipient site, whereas implant-based reconstructions do not involve a donor site, only carrying the risk of seromas at the breast itself [48]. Seromas often cause discomfort and morbidity, potentially delaying the initiation of adjuvant therapies [48]. Suction drains are typically employed at both donor and recipient sites in LDF reconstruction to help evacuate fluid and reduce seroma formation [17, 37]. However, despite drainage, seromas frequently develop after drains are removed, and opinions vary on the optimal timing for drain removal [37]. While many seromas resolve without intervention, treatment options range from pharmacologic therapy to aspiration or percutaneous drainage in an outpatient setting, classifying it in a range from CD1-3A on the Clavien-Dindo classification scale [11, 22, 23] (Table 3) (Fig. 4).

**Table 3:** Post-operative complications classifications and information

Post-operative complication	Clavien-Dindo (CD) classification	Risk factors	Mitigation strategies	Level-based evidence
Seromas	CD1-3A	–	Suction drainages [37]	III [37]
Wound dehiscence	CD1-3A	–	ciNPT [49]	II [49]
NACx necrosis	CD2-3B	Inverted T incisions with skin reducing envelope [22], NACx conservation in implant-based reconstruction [20]	Radial incisions [54]	III [54]
Flap necrosis	CD1-3B	Excessive undermining and thinning of the flaps [23] Tobacco use [22]	Smoking cessation [58] careful identification and following of the superficial fascial plane between the subcutaneous fat and mammary tissue [23]	III [23, 58]
Infection	CD1-4B	Implant-based reconstruction [12, 15, 22, 59], higher BMI, RT during or after BR [12], tobacco use [22]10/15/2025 9:01:00 AM	Prophylactic antibiotics [23]	III [23]
Capsular contracture	CD3A	Implant-based reconstruction [12, 16, 18, 19]; PMRT or prior RT [23, 24]	Anti-inflammatory substances [65]	VII [65]
Implant malpositioning	CD3B	–	ADM [66]	III [66]
Hematoma	CD3A	–	Intraoperative hemostasis [68]	VII [68]
Delayed scarring	CD1	–	Early intraoperative vasopressor use [69]	III [69]
Neurological issues	CD1	–	Nerve coaptation [70]	VII [70]
Pneumothorax	CD3B	–	Intraoperative placement of chest tube [8]	III [8]
Cystosteatonecrosis	CD1	–	Increase perforators [73]	VII [73]
Implant leak	CD3B	–	Avoidance of bras with an underwire postoperatively [76]	IV [76]

CD1 = complication that resolves on its own; CD2 = complication that only needs pharmacological intervention; CD3A = complication that requires surgical intervention but not under general anesthesia; CD3B = complication that requires surgical intervention under general anesthesia; CD4A = complication that requires admission into the ICU and is a single-system failure); CD4B = complication that requires admission into the ICU and is a multi-system failure; CD5 = complication that leads to death

Level I = systematic reviews and meta-analyses; Level II: randomized controlled trials (RCTs); Level III: cohort studies; Level IV: case-control studies; Level V: cross-sectional studies; Level VI: case reports and case series; Level VII: expert opinion

*ADM* acellular dermal matrix, *BR* breast reconstruction, *ciNPT* closed incision negative pressure therapy, *NACx* nipple-areolar complex, *PMRT* post-mastectomy radiotherapy, *RT* radiotherapy

#### Wound Dehiscence

The ACI was comparable between reconstruction groups, with 2.7% of LDF reconstructions and 2.4% of implant-based reconstructions experiencing wound dehiscence [11, 15]. The difference between the groups was not significant (*p* = 0.95). Wound dehiscence refers to the separation of a surgical wound occurring postoperatively due to various underlying causes [37]. This complication is serious and associated with high morbidity and mortality, leading to delays in recovery and increased healthcare costs [49]. Contributing factors include surgical site infections and mechanical forces that place excessive tension on the suture line [49]. A mitigation strategy that can reduce the risk of wound dehiscence is postoperative closed incision negative pressure therapy (ciNPT), which maintains a closed wound environment [49]. This promotes healing, decreases suture line tension, and provides a protective barrier against external contamination [49]. Smaller wound dehiscence may be considered a minor issue and can be managed in the clinic or office setting [12]. Similar to seromas, wound dehiscence may not always require therapy or may be treated with medications alone [22]. Based on these considerations, wound dehiscence is classified in a range from CD1-3A (Table 3) (Fig. 4).

#### Nipple-Areolar Complex (NACx) Necrosis

The ACI of NACx necrosis was 4.5% of LDF reconstruction cases compared to 6.6% in the implant-based reconstruction group [15, 20, 27]. This difference was not significant (*p* = 0.36). NACx necrosis refers to the death of NACx tissue resulting from poor vascularity and ischemia, particularly affecting the subdermal and subcutaneous layers [50, 51]. This complication leads to poorer aesthetic and patient satisfaction outcomes, including NACx flattening, hypopigmentation, deformity, and or complete loss [50, 51]. In LDF reconstruction, minimizing skin tension is critical, while in implant-based reconstruction, careful selection of implant volume is important to prevent NACx necrosis [52]. Additionally, NACx conservation during implant-based reconstruction can increase the risk of necrosis due to resection of tissue beneath the NACx that disrupts local perforators supplying blood flow [20, 53]. Incision type also plays a role with inverted T incisions with a skin-reducing envelope posing a higher risk of NACx necrosis by disrupting a larger portion of the subdermal vascular plexus [22]. However, in some cases, they may help preserve NACx vascularization [22]. Radial incisions can be a mitigation strategy by optimizing recipient vessel exposure while minimizing compromise to NACx perfusion [54]. Treatment typically involves conservative measures such as antibiotics, wound dressings, and close observation [22]. However, in more severe cases or when conservative management fails, surgical intervention becomes necessary, classifying NACx necrosis severity in a range from CD2-3B [55] (Table 3) (Fig. 4).

#### Flap Necrosis

The ACI of flap necrosis, including both total and partial flap necrosis, was similar between reconstruction groups, with 7.7% of LDF reconstructions and 7.9% of implant-based reconstructions experiencing flap necrosis (*p* = 0.98) [13, 18, 27]. Although LDF reconstruction might theoretically pose a higher risk of flap necrosis due to greater traction trauma during flap harvest and longer operative times, it is important to note that patient selection criteria for autologous breast reconstruction may contribute to this perceived risk, resulting in no observed difference in flap necrosis rates between breast reconstruction types [56]. Flap necrosis occurs when flap tissue dies because of compromised blood supply, often caused by excessive undermining and thinning of the flaps, which leads to ischemia [23]. This complication can delay the initiation of adjuvant therapies as well as impact both cosmetic outcomes and costs [57]. Known risk factors for flap necrosis include smoking and tobacco use, as nicotine and carbon monoxide in tobacco smoke are potent toxins that impair tissue oxygenation and wound healing by reducing capillary blood flow and interfering with oxygen binding [22, 58]. Because skin survival depends on maintaining well-vascularized flaps, smokers face a significant risk of flap necrosis during breast reconstruction, leading many surgeons to recommend smoking cessation prior to surgery to reduce complication rates [58]. Additional mitigation strategies include meticulous dissection along the superficial fascial plane between the subcutaneous fat and mammary tissue [23]. This allows for the preservation of critical vascularity that supplies the flap [23]. While some cases of flap necrosis may resolve spontaneously, others necessitate further surgical interventions, such as trimming necrotic tissue under local or general anesthesia to achieve wound healing [13, 20, 22, 28]. Based on these factors, flap necrosis severity is classified from CD1-3B (Table 3) (Fig. 4).

#### Infections

The ACI of infections within the LDF reconstruction group was 4% of cases compared to 9.8% of cases in the implant-based reconstruction group [12, 13, 15, 18, 20, 23, 27]. The rate of infections was significantly higher in the implant group compared to the LDF group (*p* = 0.000093). Postoperative infections can be identified in several ways, including the CDC’s criteria of the presence of purulent drainage, a positive aseptically obtained culture, peri-incisional erythema with an incision opened by the surgeon, or a physician's diagnosis of infection leading to antibiotic prescription [12]. Higher infection rates are typically seen in implant-based reconstruction, which is expected given the presence of a foreign body [12, 15, 22, 59]. Even though patients with higher BMI are indicated for autologous reconstruction, when comparing infection rates between both reconstruction types in obese patients, infection rates were found to be higher in the implant groups [60]. Infections can be classified as superficial or deep, with deep infections representing severe complications requiring hospitalization [24]. Superficial infections differ in that they do not involve periprosthetic fluid collection or drainage and usually lack systemic signs [61]. Risk factors for infection include higher BMI and RT during or after breast reconstruction [12]. A higher BMI may necessitate using a larger flap to fill a greater defect, which can stretch and compromise perforating blood vessels, thereby predisposing the flap to infection [62]. Additionally, RT can induce changes in soft tissue, including immune-mediated effects and lymphatic congestion, which both increase infection risk [63]. Interestingly, while some studies associate cigarette smoking with a higher rate of infection, others do not find a positive correlation [13, 22]. This inconsistency may be due to varying definitions of smoking across studies [64]. Smoking, as previously described, can impair oxidative bacterial killing mechanisms, leading to infection [58]. Prophylactic antibiotics are commonly used as a preventive measure before surgery, and postoperative antibiotics are also employed until signs of infection resolve [11, 23]. Treatment options for infection include explantation or implant removal in implant-based reconstruction, or debridement or flap removal in LDF reconstruction [12]. These all typically require surgical intervention under local anesthesia, although some infections may heal spontaneously [12]. Furthermore, perioperative identification of infection can sometimes halt reconstruction entirely, leaving patients without a breast at a psychologically vulnerable time [11]. In such cases, implant exchange in implant-based reconstruction carries significant risk in severe infections, whereas tissue reconstruction may provide a safer alternative for patients wishing to proceed with reconstruction [11]. LDF reconstruction may also be favored for diabetic patients at higher risk of infection-related complications [20]. Based on these considerations, infection severity is classified from CD1 to 4B (Table 3) (Fig. 4).

#### Capsular Contractures

This post-operative complication was observed exclusively in the implant-based reconstruction groups, making it a specific risk to consider when choosing this breast reconstruction type [12, 16, 18, 19]. The ACI of capsular contractures was 16% of all cases [13, 18]. 3.8% of these cases were grade II capsular contracture [17], 12% of these cases were grade III capsular contractures [13, 17], and 4.1% were grade IV capsular contractures [13]. The grading of capsular contracture is determined by the Baker score, where grades I and II reflect a normal, soft capsule, while grades III and IV correspond to a hardened, distorted breast [65]. Although a capsule naturally forms around an implant as part of the normal physiological response, capsular contracture is a complication where the capsule develops excessively into a thick, fibrous structure [65]. This condition can significantly compromise the cosmetic outcome of breast reconstruction, increasing the risk of implant malposition, leakage, and rupture, which may necessitate implant replacement [16]. Both post-mastectomy radiotherapy (PMRT) and prior RT are established risk factors for the formation of capsular contracture [23, 24]. Chronic inflammation plays a key role in its pathogenesis, suggesting that immunosuppressive strategies such as corticosteroid use might mitigate this complication [65]. However, further research is required to confirm their efficacy [65]. Treatment options for capsular contracture include capsulotomy, which may be followed by implant removal and exchange, or no additional surgery in some cases [13]. Conversion to an autologous flap, like the LDF, or the use of autologous fat transfer, can also be considered, especially in recurrent cases [21]. Additionally, capsulotomy combined with implant size reduction and lipoinjection represents an alternative approach [21]. Based on these factors, capsular contracture is classified as CD3A (Table 3) (Fig. 4).

#### Implant Malpositioning

This postoperative complication was observed exclusively in the implant-based reconstruction groups. The ACI of implant malpositioning was 3.4% of all cases [18, 20]. Implant malpositioning involves movement of the implant from its intended position within the breast pocket and can manifest as displacement, rotation, or flipping of the implant [66]. Such malpositioning can negatively impact the cosmetic outcome of breast reconstruction by causing shape or contour distortion, asymmetry, and patient discomfort [66]. One strategy to help prevent this complication is the use of an acellular dermal matrix (ADM), which can create a tighter and more stable breast pocket, reducing the risk of implant movement [66]. Specifically, in cases of IBR with implants, ADM improves implant support and reduces other complications such as capsular contracture [67]. When implant malpositioning occurs, it often necessitates implant removal, classifying it as CD3B [18, 20] (Table 3) (Fig. 4).

#### Miscellaneous

The ACI of hematoma was 4.2% in the LDF reconstruction group and 7.7% in the implant-based reconstruction group [18]. There was no significant difference between the groups (*p* = 0.6). Hematomas can develop at the breast site or at donor sites, presenting with enlargement of the affected breast and visible bruising [18, 68]. Fluid collection is particularly common with textured implants due to friction between the implant surface and the surrounding capsule [68]. Ensuring meticulous hemostasis during surgery is an important strategy to prevent hematoma formation [68]. Because hematomas often require evacuation procedures, they are classified as CD3A [20, 22].

The ACI of delayed scarring was 3% in the LDF reconstruction group and 13% in the implant-based reconstruction group [20]. The rate of delayed scarring was significantly higher in the implant group compared to the LDF group (*p* = 0.025). Delayed scarring reflects impaired wound healing and is often a consequence of radiation-induced tissue damage, regardless of the type of breast reconstruction performed [68]. This complication can impose both physical and emotional burdens on patients. A potential mitigation strategy involves the conservative use of vasopressor agents later during the procedure [69]. Although vasopressors are administered by anesthesia to manage transient periods of intraoperative hypotension, earlier use may have less impact on wound healing since adequate tissue oxygenation is critical for proper healing [69]. Treatment of delayed wound healing consists of meticulous wound care with regular dressing changes, classifying this complication as CD1 [69].

The ACI of neurological issues was 16% in the LDF reconstruction group and 8% in the implant-based reconstruction group [20]. There was no significant difference between the two groups (*p* = 0.13). Although these neurological issues were not specified, loss of breast sensation has been widely reported as a complication among post-mastectomy patients [70]. Sensation loss can be unpleasant and unanticipated for patients and may increase susceptibility to injury. Despite the higher ACI of neurological issues observed in the LDF reconstruction group, autologous breast reconstruction is generally associated with less sensory loss than implant-based reconstruction, as implants can stretch the skin and reduce the density of sensation receptors across the area [70]. A possible strategy to mitigate sensory issues is nerve coaptation, which can be performed either directly or using nerve grafts, which involves connecting severed nerves to enhance postoperative breast sensation [70]. While spontaneous sensory recovery can occur, it remains variable and unpredictable, classifying this complication as CD1 [71].

Additionally, the ACI of pneumothorax was 4% in the LDF reconstruction group and 3% in the implant-based reconstruction group, with no significant difference (*p* = 0.71) [20]. Pneumothorax is a rare but serious complication that occurs when air leaks into the pleural space due to damage or injury to the pleura, which can become life-threatening if not quickly identified and treated [8]. This complication can arise from needle puncture of the pleural cavity during either implant-based or LDF breast reconstruction procedures [8]. A potential mitigation strategy includes placing a chest tube intraoperatively if pleural injury is suspected, with the injury site subsequently repaired primarily or patched as needed, classifying pneumothorax as CD3B [8].

The ACI of cystosteatonecrosis was 4% in the implant-based reconstruction group, which occurred exclusively during lipoinjection resurfacing in the upper quadrant [21]. This complication was not reported in the autologous LDF reconstruction-only group without a prosthesis [21]. However, some studies show that reconstruction type does not significantly correlate with an increased risk of fat necrosis [72]. Also known as fat necrosis, this complication arises when breast tissue becomes injured or ischemic, leading to replacement of adipose cells with scar tissue [73]. Clinically, it can present as a palpable nodule that may be cosmetically unappealing to patients and can be mistaken for malignancy [73]. In these cases, fat necrosis resulted from fat grafting, which is a technique involving the harvest and injection of fat to correct contour deformities in breast reconstruction [73]. This is a safe and oncologically sound approach to augmenting breast volume, especially in cases where extra support may be needed [74]. A potential mitigation strategy includes increasing the number of perforators to improve perfusion to the transplanted fat tissue and reduce the risk of ischemia [73]. Despite this risk, fat necrosis often resolves over time, classifying it as CD1 [75].

The ACI of implant leak was 11.5% in the implant-based reconstruction group only [18]. Implants are not designed to last a lifetime and therefore carry a risk of rupture over time, which can lead to adverse outcomes necessitating implant removal or exchange and requiring additional surgery [76]. A possible strategy to reduce the risk of implant rupture includes avoiding the use of bras with an underwire after surgery, as limited expansibility of the lower pole can cause compression and redirect pressure toward the upper poles, increasing the likelihood of implant damage [76]. Based on these considerations, implant leakage is classified as CD3B (Table 3) (Fig. 4).

### Aesthetic Outcome

Aesthetic outcomes play a critical role in patient satisfaction following breast reconstruction, with strong correlations observed between satisfaction with the cosmetic appearance and overall functional and psychological well-being [14]. Patients undergoing LDF reconstruction generally report more favorable aesthetic experiences, particularly in terms of breast symmetry, contour, and natural appearance, as autologous reconstruction mimics the natural breast [19, 27]. These patients tend to exhibit more stable satisfaction over time and higher long-term aesthetic satisfaction, especially since implants are more likely to develop long-term complications [16, 27]. In the event of greater skin loss, delayed breast reconstruction with the LDF shows good cosmetic results [24]. The skin paddle transposed with the LD muscle can effectively replace skin deficits and cover the muscle [24, 77]. Additionally, the substantial size of the muscle flap used in LDF reconstruction can improve breast size and overall appearance, which contributes to greater patient satisfaction [27]. While immediate reconstruction with the LDF can enhance aesthetic outcomes by avoiding the immediate sense of post-mastectomy deformity, delayed reconstruction may be associated with fewer complications, which can also contribute to improved patient satisfaction [78]. In contrast, implant-based reconstruction can lead to concerns about volume, shape, or implant visibility. While initially satisfactory, long-term outcomes may show subsequent requests for size adjustments or implant replacement [21, 28]. Implants tend to maintain a fixed shape and do not adjust with the body [16]. Unilateral procedures are especially prone to developing noticeable asymmetries over time due to natural breast aging and weight fluctuations [16]. Additionally, folds and textural irregularities can be frequent with implants, which contribute to a less favorable visual outcome compared to LDF reconstruction [21]. These folds arise naturally in the implant’s outer shell due to the effects of gravity distorting it [79]. However, depending on the quality of the overlying tissue and the degree of deformation, the natural folds may become apparent on the surface of the breast [79]. Preservation of the NACx and absence of extensive scarring are also associated with greater aesthetic satisfaction across both groups, reinforcing the importance of surgical technique in shaping long-term visual and psychological results [14, 17]. RT may also be related to general and aesthetic satisfaction, as atrophy of subcutaneous fat, LD, or PM muscle, and other tissues may occur as a result of radiation [17]. This can lead to dissatisfaction with the size, shape, and touch of the breast regardless of breast reconstruction type [17]. Overall, autologous reconstruction with LDF tended to yield more durable and natural-appearing outcomes, contributing to higher patient satisfaction with body and postoperative appearance [27].

### Long-Term Functionality and Durability

In the context of a previously failed implant, LDF reconstruction is associated with a shorter recovery period and a quicker return to normal daily activities than secondary reconstruction with implants [11]. Additionally, it typically allows for a shorter duration before initiating postoperative adjuvant treatment since the use of autologous tissue is more appropriate for adjuvant treatments [27]. In contrast, implant-based reconstruction often involves complications that require additional surgical procedures, prolonging hospital stays and the recovery period [11]. The ACI of implant failures was 6.8% of cases, whereas the ACI of LDF losses was only 0.84% of cases [11, 13, 18, 20, 22, 23, 28]. The rate of implant failures was significantly higher than LDF losses (*p* = 0.0000087). Flap loss is a rare occurrence in LDF reconstruction, with minor complications being more frequent and there being less likelihood of necessitating further surgery over time [16, 38]. This favorable outcome is attributed to the LDF’s well-vascularized tissue and robust vascular supply, although partial flap loss does occur in some cases [18, 38]. Implant-based reconstruction is more likely to develop long-term complications with developments such as scar tissue formation leading to capsular contracture, malposition, leakage, and rupture [16]. These complications stem from the body’s reaction to the implant as a foreign device [16]. Implant-related complications often lead to implant removals and additional surgeries, with secondary breast reconstruction occurring as late as 8 years postoperatively [13, 16]. Implant removals can also occur because of infection, recurrent disease, implant exposure, necrosis, and severe or chronic pain [13, 15, 18, 20, 23, 27]. Notably, in cases of implant failure, LDF reconstruction is often indicated as a salvage option, while the reverse is uncommon [11]. While implant exchanges may occur, some patients undergo implant removal without further reconstruction, whereas LDF reconstruction has a lower rate of reconstructive abandonment [18]. Despite these benefits, LDF reconstruction is more often associated with additional axillary procedures, particularly related to the sentinel node [20]. This is because patients with cancer or advanced tumor stages are often indicated for LDF reconstruction to facilitate appropriate adjuvant treatments [20]. Both LDF and implant-based reconstructions carry risks of shoulder morbidity [26]. However, in LDF reconstruction procedures, detachment of the LD muscle from its origin and its transfer to the breast site can result in significant loss of shoulder function [26]. Implant-based reconstruction can also affect shoulder mobility as implant placement may increase tension on the PM muscle and tendon, leading to tightness that can impair rehabilitation and scapulothoracic motion [26]. However, LDF reconstruction may result in lower mean flexion and abduction of the shoulder due to the involvement of a large muscle like the LD [26]. Both breast reconstruction types can also affect the physical well-being of the chest [15, 25]. Implants may cause discomfort, such as tightness and pain, due to their placement either above or beneath the PM muscle [25]. However, LDF may involve more perioperative pain due to the extensive muscle dissection required during surgery [14]. Although both breast reconstruction types alter the chest, patients’ perception of their breast reconstruction outcomes and their willingness to undergo the same procedure again depend on multiple factors, including satisfaction with presurgical medical consultation [14].

### Psychological Impact

LDF reconstruction is associated with an overall higher satisfaction rate compared to implant-based reconstruction [11, 15, 20]. This satisfaction extends to various aspects of patients’ lives, including sexual and social well-being, body image, breast shape, appearance in fitted clothing, bra fit, softness, sizing or symmetry, naturalness, and overall contentment with the results [11, 14, 25]. Achieving a more natural-looking breast through autologous reconstruction, which mimics the natural breast, can significantly enhance sexual well-being and satisfaction with the breast’s natural appearance [19]. Using autologous tissue also helps maintain breast softness, allowing it to conform better to bras and fitted clothing, which can improve body image and boost self-confidence, positively influencing other aspects of life. Regarding nipple reconstruction, patients who undergo LDF reconstruction often report higher satisfaction in terms of nipple shape, appearance, naturalness, color, and projection [25]. This may be attributed to the well-vascularized and perfused tissue bed provided by the LDF, which supports nipple reconstruction and contributes to superior psychosocial well-being and therapeutic benefits [15, 27, 38]. Conversely, patients receiving implant-based reconstruction may experience heightened stress or psychological difficulties if multiple procedures become necessary due to complications [11, 14]. As previously noted, implant complications are more likely to require further surgeries, whereas LDF-related complications are generally minor and often managed non-surgically [13, 16, 38]. Additionally, anxiety related to implant-based reconstruction may be exacerbated by concerns over implants being foreign bodies, potentially leading to less satisfaction with sexual confidence and experiences when nude [25, 28]. This is in comparison to LDF reconstruction, which can produce a more natural appearance to the breast [19]. Nevertheless, implant-based reconstruction may be perceived as less burdensome than LDF reconstruction as it avoids an additional donor site and is therefore less invasive [14]. This can enhance sexual confidence in patients who feel reassured by the absence of a large donor site scar and improved physical function [14]. Experiences of pain also vary between procedures, with some patients reporting greater perioperative pain and movement restriction with LDF reconstruction due to the involvement of a large muscle like the LD [14]. Others find implants to be less painful overall, a perspective often echoed by medical and paramedical teams [14, 20, 25]. Despite LDF reconstruction generally offering superior overall satisfaction, implants can still leave many patients equally satisfied with their results [17, 23–25]. Other factors, such as patient age and PMRT, play a role in breast satisfaction [25]. Older age tends to be a risk factor for more complications, and PMRT can be a risk factor in patients receiving implant-based reconstruction [13, 15, 18, 19, 22]. Overall, breast reconstruction after mastectomy can be a fearful experience for patients, especially the idea of further surgery and hospitalization. However, breast reconstruction is primarily performed to restore physical appearance, and many patients experience rapid psychological and social reintegration after the procedure [15, 28].

### Revisions

LDF reconstruction is less likely to require revision surgery compared to implant-based reconstruction [11, 15, 16]. The ACI of revisions among LDF reconstruction was 0% while 80.5% of implant-based reconstruction required additional procedures [11, 15, 16]. The rate of revisions in the implant group was significantly higher compared to the LDF group (*p* = 0). This difference arises because autologous techniques like LDF reconstruction use the patient’s own tissue, allowing the reconstructed breast to age and adapt to weight changes over time, similarly to the natural breast [16]. In contrast, implants maintain a fixed shape and do not adjust with the body [16]. Additionally, because implants are foreign bodies, they carry an increased risk of complications that can ultimately necessitate replacement [16]. When implants fail, revision often involves implant exchange or conversion to LDF reconstruction [11, 13, 20, 21]. This is because LDF reconstruction offers a safe procedure following complications, avoiding further issues and longer durations of care [11]. However, some patients may choose removal of the implant without undergoing further reconstruction to avoid additional surgeries and the potential complications associated with autologous reconstruction [11, 18]. Importantly, the need for revision surgery is influenced by more than the type of breast reconstruction. Factors such as patient age, ethnicity, geographic region, receipt of chemotherapy (CT), existing comorbidities, and timing of the initial surgery can all impact the likelihood of requiring revisions [16, 18, 20].

### Limitations

Firstly, this systematic review is limited by the retrospective nature of the included studies inherently introduces biases, such as selection bias and inconsistent reporting standards, which may affect the strength of the conclusions drawn. Additionally, the large number of articles reviewed increases the potential for human error during the data extraction and synthesis process, despite efforts to minimize discrepancies through independent review and consensus. As with any systematic review, variability in study design, patient populations, and outcome measures across the included studies further complicates direct comparisons between reconstruction techniques. These factors should be considered when interpreting the findings of this review. Lastly, differences in study design and patient populations across the articles make comparisons challenging and should be kept in mind when interpreting the results.

## Conclusion

LDF reconstruction generally offers more natural aesthetic outcomes and higher patient satisfaction with fewer long-term revisions, though it involves longer operative times, potential donor site morbidities, and more postoperative complications. In contrast, implants provide a less invasive option with fewer postoperative complications but are associated with less satisfaction and higher rates of revision surgeries over time. Though LDF reconstruction involves more complications, there are far fewer long-term consequences compared to implant-based reconstruction, which can be prone to issues such as folds, volume concerns, and asymmetry. Indications and contraindications for both breast reconstruction types should be addressed preoperatively to minimize complications. Ultimately, reconstruction choice should be individualized, considering aesthetic goals, surgical risk, and patient preferences to optimize long-term satisfaction and quality of life. Surgeons must take a comprehensive, individualized approach when advising patients, considering factors such as aesthetic goals, tolerance for multiple surgeries, underlying risk profiles, and patient preferences.

## Supplementary Information

Below is the link to the electronic supplementary material.

Supplementary file1 (DOCX 43 kb)
