# Supplementary material for: Comparing Latissimus Dorsi Flap to Implant in Breast Reconstruction Following Mastectomy in Breast Cancer Patients: A Systematic Review
Source: Aesthetic Plast Surg. 2025 Oct 29;50(5):1776–96. doi: 10.1007/s00266-025-05335-4 (PMC13009031; doi:10.1007/s00266-025-05335-4)
Supplement: Supplementary file 1 — Supplementary file1 (DOCX 43 kb) [file 266_2025_5335_MOESM1_ESM.docx]

**Supplementary Table 1: Search Strategy**

| Search strategy | Pubmed | ((("Breast Implants"[Mesh] OR Breast Implants OR "Breast Implantation"[Mesh] OR Breast Implantation) AND (Latissimus Dorsi Flap Breast Reconstruction OR Latissimus Dorsi Flap Reconstruction OR LD Flap Reconstruction OR Latissimus Dorsi Breast Reconstruction OR Latissimus Dorsi Tissue Transfer OR Latissimus Dorsi Muscle Flap OR LD Flap Surgery OR Back Flap Reconstruction OR Latissimus Dorsi Myocutaneous Flap OR LD Breast Reconstruction OR Latissimus Dorsi Flap Breast Surgery OR Latissimus Dorsi Autologous Reconstruction OR Latissimus Dorsi Graft OR Latissimus Dorsi Flap Procedure OR Latissimus Dorsi Flap Transfer OR Muscle Flap Breast Reconstruction OR Latissimus Dorsi-Based Reconstruction OR Back Tissue Flap Reconstruction OR Latissimus Dorsi Surgical Reconstruction)) AND ("Mastectomy"[Mesh] OR Mastectomy OR "Mastectomy, Simple"[Mesh] OR Mastectomy, Simple)) AND ("Breast Neoplasms"[Mesh] OR Breast Neoplasms OR breast cancer) |
| --- | --- | --- |
|  | Cochrane | (Breast implant OR breast prosthesis OR breast prosthetic OR silicone implant OR saline implant OR breast prosthetic device OR breast augmentation implant OR implantable breast prosthesis OR mammary implant OR cosmetic breast implant OR silicone breast prosthesis OR saline breast prosthesis OR breast augmentation device OR implantable breast device OR breast reconstruction implant OR implanted breast prosthesis OR implanted breast device OR breast filler implant OR mammary prosthesis OR implantable mammary prosthesis OR implant-based augmentation OR breast volume implant) AND (Latissimus Dorsi Flap Breast Reconstruction OR Latissimus Dorsi Flap Reconstruction OR LD Flap Reconstruction OR Latissimus Dorsi Breast Reconstruction OR Latissimus Dorsi Tissue Transfer OR Latissimus Dorsi Muscle Flap OR LD Flap Surgery OR Back Flap Reconstruction OR Latissimus Dorsi Myocutaneous Flap OR LD Breast Reconstruction OR Latissimus Dorsi Flap Breast Surgery OR Latissimus Dorsi Autologous Reconstruction OR Latissimus Dorsi Graft OR Latissimus Dorsi Flap Procedure OR Latissimus Dorsi Flap Transfer OR Muscle Flap Breast Reconstruction OR Latissimus Dorsi-Based Reconstruction OR Back Tissue Flap Reconstruction OR Latissimus Dorsi Surgical Reconstruction) AND (Mastectomy OR breast removal surgery OR breast tissue removal OR total mastectomy OR simple mastectomy OR radical mastectomy OR modified radical mastectomy OR partial mastectomy OR bilateral mastectomy OR subcutaneous mastectomy OR mastectomy procedure OR breast cancer surgery OR mastectomy operation OR breast excision) AND (breast cancer OR mammary carcinoma OR breast carcinoma OR malignant breast tumor OR breast malignancy OR mammary cancer OR carcinoma of the breast OR malignant mammary neoplasm OR ductal carcinoma OR lobular carcinoma OR invasive breast cancer OR non-invasive breast cancer OR metastatic breast cancer OR breast neoplasm OR breast adenocarcinoma OR breast sarcoma OR mammary neoplasm OR breast lesion) |
|  | Web of Science | (((ALL=(Breast implant OR breast prosthesis OR breast prosthetic OR silicone implant OR saline implant OR breast prosthetic device OR breast augmentation implant OR implantable breast prosthesis OR mammary implant)) AND ALL=(Latissimus Dorsi Flap Breast Reconstruction OR LD Flap Reconstruction OR Latissimus Dorsi Breast Reconstruction OR Latissimus Dorsi Tissue Transfer OR Latissimus Dorsi Muscle Flap OR LD Flap Surgery OR Back Flap Reconstruction)) AND ALL=(Mastectomy OR breast removal surgery OR breast tissue removal OR total mastectomy OR simple mastectomy OR radical mastectomy OR breast excision)) AND ALL=(breast cancer OR mammary carcinoma OR breast carcinoma OR malignant breast tumor OR breast malignancy OR mammary cancer OR carcinoma of the breast OR malignant mammary neoplasm OR invasive breast cancer OR non-invasive breast cancer OR metastatic breast cancer OR breast neoplasm OR breast adenocarcinoma OR mammary neoplasm) |
|  | VHL | Breast implant OR breast prosthesis or breast prosthetic AND Latissimus Dorsi Flap Breast Reconstruction OR LD Flap Reconstruction OR Latissimus Dorsi Breast Reconstruction AND Mastectomy OR breast removal surgery OR simple mastectomy AND breast cancer OR mammary carcinoma or malignant breast tumor or breast malignancy OR mammary cancer |
| Date initiated | 1/22/2025 | |
| Initial findings | Total = # 785   - PubMed: # 371 - Cochrane: # 37 - VHL: # 41 - WOS: # 336 | |
| Removed before screening (Duplicates) | Excluded = 155  Included = 630 | |

**Supplementary Table 2: Summary of Outcomes**

| Author, Year of Publication | Indication | Contraindication | Technique | Postoperative Complications | Risk Factors | Long Term Functional Outcome and Durability | Psychological Impact | Revisions | Mortality |
| --- | --- | --- | --- | --- | --- | --- | --- | --- | --- |
| Allweis et al., 2002 [11] | - | - | Implant: TE later substituted with an implant | - | - | Both:  - Days to starting adjuvant CT later in no BR group than BR group | - | - | - |
| Asal et al., 2024 [12] | - | - | LDF:  - Used transverse or oblique LD based on available tissue  Implant: Exchange included immediate implant removal and exchange with a TE followed by DBR with silicon or IBR with a smaller size silicone implant | LDF:  - 7 (38.9%) seromas requiring aspiration  - 2 (11.1%) wound dehiscence | - | LDF:  - Had shorter recovery period  - Quicker return to normal daily activities  Implant:  - Had longer recovery period, hospital stay, and more surgical procedures  - 4 (28.6%) implant failures that were converted to LDF  - 3 (21.4%) implant removals with no further BR | LDF:  - Had higher overall satisfaction  - Increased satisfaction rate in sexual life  - Increased satisfaction rate in social life  - Increased satisfaction rate in overall results  Implant: Had higher stress due to multiple procedures and complications | LDF: No revision surgery  Implant:  - 7 (50%) revisions  - 4 (28.6%) implant failures that were converted to LDF  - 3 (21.4%) implant removals with no further BR | - |
| Bennett et al., 2018 [13] | - | - | - | LDF:  - 28 complications (39.4%)  - 10 (14.1%) reoperative complications  - 6 (8.5%) wound infections  Implant:  - 441 (26.9%) complications  - 258 (15.8%) reoperative complications  - 176 (10.8%) wound infections | Both:  - Older age and current smoking associated with increased odds of any complications or reoperative complications  - Higher BMI and RT during or after BR associated with increased odds of any complications, reoperative complications, and wound infection  - NAC and adjuvant CT associated with increased odds of reoperative complications | LDF: 2 (2.8%) failures  Implant: 116 (7.1%) failures | - | - | - |
| De Lorenzi et al., 2010 [14] | - | Implant: Preoperative RT | Implant: Anatomical shaped silicone implant and round-shaped silicone in various sizes used as well as TE in some cases | Implant:  - 3 (6.12%) infections  - 3 (6.12%) partial necrosis mastectomy flaps leading to 1 (2.04%) exposure  - 2 (4.08%) grade III capsular contractures  - 2 (4.08%) grade IV capsular contractures | Both: Cigarette smoking positively correlated to early complications | Implant:  - 3 (6.12%) removals due to late infection  - 1 (2.04%) removal due to exposure  - 2 (4.08%) late removals due to Baker III-IV capsular contracture with 1 receiving implant change | - | Implant:  - 7 (14.3%) implant removals  - 1 (2.04%) removal that had implant change | - |
| Ditsch et al., 2013 [15] | - | - | Implant: Sub- or pre-pectoral replacement of prostheses with or without prior  use of TE | Both:  - Complications included infection, hematoma, hemorrhage, necrosis or seroma | - | Both: Significant correlation between higher consent rate to re-reconstruction and positive first preoperative counseling  LDF: More perioperative pain and movement restriction than implant | Both: Significant correlation between patient satisfaction with presurgical medical consultation and whether they would choose the same BR again with many stating that they would do so  LDF:  - Better body image outcomes  Implant:  - Considered least burdensome method  - Better sexuality than LDF  - Association with worse psychological adjustment and more chronic pain  - 13 (54.2%) stated no pain  - 39 (44%) unsatisfied  - Rated 10.95/30 body image | - | - |
| Gao et al., 2022 [16] | LDF:  - Axillary lymph node metastasis with a high likelihood of postoperative RT  - Large tumors with a strong desire to conserve the breast  - High tumor/breast ratios but small breast size | Implant: Contraindications to porcine-based products excluded from the SIS matrix-assisted DTI group | Implant:  - Inferior and outer origins of the PM muscle are released to create a subpectoral pocket and then a definitive implant is placed below the muscle  - SIS matrix is fixed to the chest wall to cover and support the lower and lateral areas (close the pocket) of the implant according to the insufficiency of the PM muscle | LDF:  - 5 (8.9%) major complications that required rehospitalization or reoperation  - 6 (10.7%) minor complication managed with dressings or medication  - 0 (0%) infectious complications  - 6 (10.7%) seromas  - 0 (0%) dehiscence  - 0 (0%) NACx necrosis  Implant:  - 5 (6.3%) major complications  - 9 (11.4%) minor complications  - 4 (5.1%) infectious complications  - 6 (7.6%) seromas  - 2 (2.5%) dehiscence  - 2 (2.5%) NACx necrosis | - | LDF:  - 1 (1.8) reported chronic pain  - 4 (7.1%) tumor metastasis  Implant:  - 2 (2.5%) reported chronic pain  - 5 (6.3%) implant loss  - 0 (0%) tumor metastasis  - Superior physical well-being of the chest and satisfaction with the surgeon | LDF:  - Superior psychosocial well-being rated 84.31/100  - Superior sexual well-being rated 69.65/100  - Superior satisfaction with the breast and satisfaction with information  Implant:  - Lower rated psychosocial well-being 73.52/100  - Lower rated sexual well-being 50.95/100 | Implant: 5 (6.3%) implant losses requiring secondary revision operations | - |
| Johnson et al., 2023 [17] | - | - | Implant: Two-stage TE-implant procedure was used at the beginning of the study and single-stage DTI procedure was used later | - | Both:  - IBR more recently is more likely to undergo secondary BR  - Women in the Northeast, Southeast, and West Midlands more likely to undergo secondary BR at 3 years than those in London  - Women in the Southeast and West Midlands more likely to undergo secondary BR at 5 years and 3 years respectively  Implant:  - In addition to implant-based primary BR, younger age, invasive versus preinvasive disease, and receipt of CT were strongly associated with increased rates of secondary BR  - Nodal involvement strongly associated with increased secondary BR at 3 and 5 years | LDF: Unlikely to require any further surgery over time  Implant:  - More likely to undergo secondary BR with 663 (12.8%) at three years, 535 (14.3%) at five years, and 189 (17.6%) at eight years  - More likely to develop complications over time, including the development of scar tissue leading to capsular contracture, malposition, leakage, and rupture | - | Both:  - Strong association between receipt of one or more revisions and the patient's age and the type of BR performed  - Strong association also between ethnicity, region, receipt of CT, and Charlson Co-morbidity Index score and the receipt of one or more revisions at 3 and 5 years  - Cancer-related factors including disease status and nodal involvement not associated with revisional surgery  - Women undergoing BR more recently were less likely to have undergone one or more revision than those who had surgery earlier  LDF: Had more patients with no revisions at all 3 time points  Implant:  - More likely to require revision and the odds increasing with time  - 3147 (60.6%) underwent at least one revision by three years  - 747 (69.5%) underwent at least one revision by eight years  - 349 (32.5%) required two or more revisions by eight years | - |
| Lei et al., 2020 [18] | LDF: Generally older and higher BMI  Implant: Younger patients with lower BMI | - | - | - | - | - | Both: No significant satisfaction differences were found between implant and autologous reconstruction groups | - | - |
| Lipa et al., 2003 [19] | LDF:  - After failed implant  - Preoperative RT and use of autogenous tissue  Implant: Thinner patients with smaller breast volumes or those undergoing bilateral BR | - | LDF:  - 1 (4.2%) involved an endoscopically harvested muscle-only flap  - 13 (54.2%) involved LDF  - 10 (41.7%) involved extended LDF  Implant:  - 18 (69.2%) used TE followed by permanent implant placement  - 8 (30.8%) were single-stage | Both: Some perioperative medical complications  LDF:  - 10 (41.7%) breast-site complications and 9 (37.5%) donor-site complications  - 2 (8.3%) medical complications without long-term sequelae  - 1 (4.17%) hematomas  - 7 (29.17%) seromas  - 4 (16.67%) infections  - 5 (20.83%) mastectomy flap necrosis  - 3 (12.5%) wound healing complications  Implant:  - Complications more frequent with 20 (76.9%) complications  - 2 (7.68%) hematomas  - 4 (15.38%) seromas  - 6 (23.08%) infections  - 4 (15.38%) mastectomy flap necrosis  - 2 (7.69%) implant mispositioning  - 3 (11.54%) implant leaks  - 8 (30.77%) capsular contractures (7 were grade III and 1 was grade II) | Both:  - Age alone should not determine BR type  - Current smoking, IBR, and higher BMI increased breast-site complications  Implant: Complication rates higher among older women, especially implant-based BR | Both:  - Some exhibited no evidence of disease during follow-up of 4.2 years  - Some experienced at least one breast-related complication with the most common being mastectomy flap necrosis  LDF:  - No abandoning of reconstructive efforts  - 21 (86.3%) five-year survivals  - 1 (4.2%) partial flap loss  - 0 (0%) complete flap losses  Implant:  - 11 (42.3%) total removals  - 3 (11.5%) removals due to recurrent disease or uncontrolled infection  - 1 (3.8%) removal due to mastectomy flap necrosis resulting in exposure and requiring a skin graft for closure  - 3 (11.5%) removals due to leaks  – 2 (7.7%) removals due to Baker III capsular contracture  - 1 (3.8%) removals due to implant mispositioning and dissatisfaction with the feel and appearance of the saline-filled implant  - 1 (3.8%) removal due to severe pain resulting in an inability to tolerate the expansion process  - 1 (3.8%) removal due to refusal of the silicone  - 7 (28%) removals without further procedures  - 24 (90.9%) ten-year survivals | - | Implant: Significantly greater mean number of additional operations performed to complete reconstruction | - |
| Liu et al., 2020 [20] | LDF: Small breast size | Implant:  - IBR (single-stage) and adjuvant RT | - | - | Implant: RT increases complications for implants | - | - | - | - |
| Mazard et al., 2024 [21] | LDF:  -Mastectomies performed due to cancer diagnosis  - Higher risks of complications related to scarring and infection  - Higher BMI  Implant: Donor site is not sufficient (lower BMI) | - | Both: TE used when skin surface was not sufficient in the opinion of the surgeon to avoid tissue damage  LDF: 18 (20%) adipose tissue transfers  Implant:  - 42 (66%) used a retro-pectoral implant  - 3 (5%) adipose tissue transfers | Both:  - No severe complications (greater than grade 4)  LDF:  - 36 (39%) incidences  - 14 (15%) seromas with 10 (11%) requiring and 4 not requiring drainage with a median of 1 puncture  - 6 (7%) infections  - 9 (10%) NACx skin necrosis  - 3 (3%) delayed scarring  - 15 (16%) neurological issue  - 4 (4%) pneumothorax  Implant:  - 39 (62%) incidences  - More grade 3 complications with 32 (50.8%) grade 3b complications  - 7 (11%) seromas with 3 (5%) requiring and 4 (6%) no requiring drainage with a median of 0.4 punctures  - 7 (11%) NACx skin necrosis  - 4 (6%) hematomas  - 8 (13%) delayed scarring  - 5 (8%) neurological issue  - 2 (3%) pneumothorax  - 1 (2%%) implant rotation | Both:  - BMI impacted incident rate with risk increasing from 24.5 and higher  - Smoking status and diabetes did not impact incidences, but patients were encouraged to stop smoking from their first visit and surgery was postponed until patient stopped to minimize risk  Implant:  - Higher rate of NACx conservation could lead to increased necrosis risk | Both: Arm mobility following surgery was not ignorantly impacted  LDF:  - Shorter hospital stays  - 73 (79%) associated with an additional axillary procedure  Implant:  - 11 (17%) with greater than six days in the hospital  - Higher use of morphine in about over 32 (50%) patients  - 4 (6.3%) evacuations of hematomas  - 2 (3.2%) surgical trimmings due to necrosis  - 6 (9.5%) implant ablation due to necrosis  - 1 (1.6%) implant ablation due to implant rotation  - 1 (1.6%) switch to LDF | LDF:  - Higher satisfaction rate  Implant:  - Considered less painful according to medical and paramedial teams  - Shorter maximum recorded pain during hospital stay with only 3 (4.8%) reporting a visual analogue scale score greater than 8 | Implant:  - Second surgery was required more often  - Average of 1-2 revision surgeries  - 18 (28.6%) grade 3b patients required revision surgery due to disappointing cosmetic outcome  - 28 (44.4%) requested revisions to modify implant  - 1 (1.6%) switch to LDF | Both: No patient death |
| Missana et al., 2007 [22] | - | - | Both: Lipofilling performed in all cases to provide correction | Both: No cases of microcalcifications suggestive of malignancy  Implant: 1 (4%) cystosteatonecrosis in the upper quadrant during lipoinjection resurfacing | - | - | - | Both: All cases received lipofilling  Implant: 9 (36%) implant volumes changed with lipoinjection | - |
| Quilichini et al., 2020 [23] | LDF: High BMI, high breast cup-size, neoadjuvant RT, and local recurrence | - | LDF: Some procedures performed robotically | Both:  - Complications ranged from grade 1-4 and included skin and/or NACx suffering or necrosis, and hematoma  LDF:  - 14 (9.7%) grade 2-4 complications  - Most grade 1 complications were dorsal seromas  - Grade 2 were dorsal seromas and hyperthermia  - Grade 3 were hematomas, infection, and partial LDF necrosis  Implant: 55 (10%) grade 2-4 complications | Both:  - Grade 2-3 complications significantly associated with smoking tobacco  - Complications also increased by high breast cup-size and IBR type  Implant: Loss rates significantly associated with type of complication | LDF: Grade 3 complications required reoperation  Implant: 26 (4.7%) implant losses | - | - | - |
| Reefy et al., 2010 [24] | Implant: High risk of requiring PMRT | - | - | Both:  - No wound complications  - Some developed distant disease  LDF: 1 (100%) developed donor-site seroma  Implant:  - 2 (3.9%) infections  - 1 (2%) smoker developed marginal skin envelope ischemia | Implant: PMRT or prior RT had more significant capsule formations requiring capsulotomy | LDF:  - No local recurrences  - No partial or total flap losses  - 1 (100%) developed donor-site seroma requiring percutaneous drainage in the outpatient setting  Implant: 2 (3.9%) infections requiring implant removal | Both: High levels of patient satisfaction | - | Both:  - 1 patient death by lung cancer  - 1 by metastatic breast cancer |
| Sanguinetti et al., 2016 [25] | - | Implant: After irradiation | - | Both: Presence of local complications in many cases with the most common being seroma followed by superficial infection  LDF: 16.7% of LDF complications were seromas  Implant:  - Lowest prevalence of complications only 4 (16.7%) having superficial infection and the absence of readmission  - Local complications included capsular contracture (Baker III/IV) which was the 2nd most frequent complication overall  - Deep infection in 22.2% of permanent TE complications  - 16.7% of single-step implant complications  - Deep infection and extrusion required all patients to be hospitalized for treatment | Both:  - Operative time, timing of BR, and type of adjuvant treatment not correlated with incidence of complications  - Intrinsic difficulty of procedure is associated with incidence of complications  - Incidence of complications was lower in patients undergoing BR delay without RT and higher in those undergoing DBR in combination with RT  Implant: Capsular contracture has an increased incidence when receiving RT after implant | Both: No significant difference in length of stay between LDF and implant  Implant: Single-step implant had the highest incidence of readmission with 7 (33.3%) | Both: BR provides satisfactory results | - | Both: No patient deaths |
| Tomita et al., 2023 [26] | - | - | Implant: Inserted beneath PM muscle | - | - | Both:  - No significant difference in physical well-being of chest between groups  - Age, PMRT, and breast size associated with physical well-being of chest  LDF: Associated with physical well-being of the chest | Both:  - No significant difference between reconstruction types in patients' willingness to make the same choice again or whether they regretted having surgery, as well as pain in the breast area, satisfaction with the nipple reconstruction, and satisfaction with the outcomes  - Age and PMRT associated with breast satisfaction  LDF:  - Satisfaction with the breast was superior to implant-based BR in terms of shape, with fitted clothing, lining up, bra fit, softness, equal sizing, natural look, natural hang, and feeling to touch  - Associated with breast and nipple reconstruction satisfaction  - Satisfaction with the nipple reconstruction superior to implant-based BR in terms of shape, appearance, natural look, color, and nipple projection  - Superior to implant in terms of meeting expectations perfectly  - Achieved higher average psychosocial and sexual well-being scores than implant but no significant difference  Implant:  - Lower score in patient satisfaction with their sex life and their sexual confidence when nude  - Often causes discomfort such as tightness and pain | - | - |
| Woo et al., 2018 [27] | - | - | LDF:  - LD muscle with overlying abundant adipose tissue and skin paddle was elevated and transferred to the mastectomy site with humeral insertion of the detached muscle  - Thoracodorsal nerve was excised at 1 cm width to prevent involuntary muscle movement  - Two suction drainages inserted into mastectomy site  Implant:  - Implant inserted into subpectoral pocket with or without acellular dermal matrix  - Two drainages inserted with one for subpectoral space and one for prepectoral space | - | Both:  - Old age, presence of tumor-positive lymph nodes, and NAC were independent risk factors for the development of shoulder morbidity  - Early rehabilitation with two postoperative months reduced the risk of sustained shoulder morbidity with late rehabilitation timing showing a significantly increased rate of sustained shoulder morbidity  - ALND was an independent predictor for developing sustained shoulder morbidity  - Tumor staging and adjuvant oncologic treatments did not influence the development of sustained shoulder morbidity | Both:  - No difference among the reconstruction groups in terms of the onset and duration of shoulder morbidity  - Number of patients with complete recovery was similar across all groups  LDF:  - Is a significant risk factor for shoulder morbidity with it being the most common with 19 (43.2%) cases  - Lower mean flexion and abduction than implant group  Implant: Is a significant risk factor for shoulder morbidity with 53 (23.8%) cases of shoulder morbidity | - | - | - |
| Xia et al., 2023 [28] | - | Implant: Sentinel lymph node metastasis | LDF:  - Lymph node biopsy first  - Subcutaneous gland resection same as implant  - Skin was cut according to preoperative design incision and the skin flap was separated  - To select the LD with adipose tissue, the tissue was separated to the scapula, a subcutaneous tunnel was established, and the back bleeding was stopped  - To fix LDF around chest wall, breast plasticity was performed, bleeding was stopped fully, a drainage tube was placed, and the incision after the flap was free of ischemia was closed  Implant:  - Silicone prostheses prepared according to the patient's breast size and sentinel lymph node status  - A subcutaneous gland resection was performed from the areola edge to the outside of the mammary gland, and thin layers of tissue were reserved to ensure blood supply of the nipple  - The PM fascia was preserved but then partially stripped to remove most of the PM origin  - A drainage tube was placed, prosthesis was gradually implanted and retained for drainage, PM fascia was sutured and wrapped, and then incision was closed | LDF:  - Safer than implant with only 5 (9.62%) complications  - 2 (3.85%) hematomas/seromas  - 2 (3.85%) wound infections  - 1 (1.92%) flap necrosis  - 0 (0%) NACx necrosis, dilator disorder, fat liquefaction, and abdominal hernia  Implant:  - 12 (30.77%) complications  - 3 (7.69%) wound infections  - 3 (7.69%) NACx necrosis  - 2 flap necrosis  - 4 (10.26%) prosthetic disorders  - 0 (0%) hematomas/seromas, dilator dysfunctions, fat liquefaction, and abdominal hernias | Both: BMI, intraoperative bleeding volume, and BR type are independent risk factors that affect the efficacy and safety of postoperative BR for cancer | Both:  - No dramatic difference in postoperative drainage time and rehabilitation rate between BR types  - Significant difference in local recurrence rate at 1 day versus 30 days after surgery  - Significant difference in distant metastasis rate of breast cancer in the 40-49-year-old age group  LDF: Time of postoperative adjuvant therapy was dramatically shorter than in implants  Implant: Over time, the local recurrence rate gradually increased compared to LDF with a significant difference at 12 days | LDF:  - Exhibited better therapeutic effects  - 7 and 30 days after surgery, had higher patient satisfaction than implant | - | - |
| Zekri et al., 1996 [29] | LDF: Small to medium breast patients who did not want prosthetic reconstruction because of foreign body concerns  Implant:  - T1-T2 tumors in patients with small to medium breasts who had adequate skin and intact healthy PM  - Mammary TE indicated for medium to large breast who had tight skin after the mastectomy and did not want additional scars | - | Implant: Used textured saline filled mammary prosthesis or mammary TE | - | - | Both:  - No complications related to BR with adjuvant therapy  - Some patients opted for NACx reconstruction within 6 months of BR  - No recurrence after 2 years follow-up  Implant: 1 (5.26%) implant removal | Both:  - Many patients displayed hesitancy and initial fear of further surgery and hospitalization after mastectomy  - Majority of patients expressed positive feelings, both physically and regarding their sexuality, with rapid psychological and social re-integration  LDF: All patients expressed extreme satisfaction  Implant:  - 13 (92.3%) patients expressed extreme satisfaction  - 1 (5.26%) had severe anxiety and phobia about implant leading to removal of the prosthesis | - | - |

ALND = axillary lymph node dissection; BMI = body mass index; BR = breast reconstruction; CT = chemotherapy; DBR = delayed breast reconstruction; DTI = direct-to-implant; IBR = immediate breast reconstruction; LD = latissimus dorsi; LDF = latissimus dorsi flap; NAC = neoadjuvant chemotherapy; NACx = nipple-areolar complex; PM = pectoralis major; PMRT = post-mastectomy radiotherapy; RT = radiotherapy; TE = tissue-expander
